# The JCSG high-throughput structural biology pipeline

**DOI:** 10.1107/S1744309110038212

**Published:** 2010-09-30

**Authors:** Marc-André Elsliger, Ashley M. Deacon, Adam Godzik, Scott A. Lesley, John Wooley, Kurt Wüthrich, Ian A. Wilson

**Affiliations:** a Joint Center for Structural Genomics (JCSG), http://www.jcsg.org, USA; b Department of Molecular Biology, The Scripps Research Institute, La Jolla, CA, USA; c Stanford Synchrotron Radiation Lightsource, SLAC National Accelerator Laboratory, Stanford University, Menlo Park, CA, USA; d Program on Bioinformatics and Systems Biology, Sanford–Burnham Medical Research Institute La Jolla, CA, USA; e Center for Research in Biological Systems, University of California, San Diego, La Jolla, CA, USA; f Genomics Institute of the Novartis Research Foundation, San Diego, CA, USA

**Keywords:** structural genomics, Joint Center for Structural Genomics, Protein Structure Initiative

## Abstract

The Joint Center for Structural Genomics high-throughput structural biology pipeline has delivered more than 1000 structures to the community over the past ten years. The JCSG has made a significant contribution to the overall goal of the NIH Protein Structure Initiative (PSI) of expanding structural coverage of the protein universe, as well as making substantial inroads into structural coverage of an entire organism. Targets are processed through an extensive combination of bioinformatics and biophysical analyses to efficiently characterize and optimize each target prior to selection for structure determination. The pipeline uses parallel processing methods at almost every step in the process and can adapt to a wide range of protein targets from bacterial to human. The construction, expansion and optimization of the JCSG gene-to-structure pipeline over the years have resulted in many technological and methodological advances and developments. The vast number of targets and the enormous amounts of associated data processed through the multiple stages of the experimental pipeline required the development of variety of valuable resources that, wherever feasible, have been converted to free-access web-based tools and applications.

## Abstract 2

The Joint Center for Structural Genomics high-throughput structural biology pipeline has delivered more than 1000 structures to the community over the past ten years and has made a significant contribution to the overall goal of the NIH Protein Structure Initiative (PSI) of expanding structural coverage of the protein universe.

## 1. Structural genomics: evolution of structural biology

The landscape of structural biology has changed dramatically during the past decade. At the turn of this century, technological advances broadened the application of structural biology to a much larger audience by increasing the number and the complexity of specific biological problems that could be addressed. This evolution in molecular biology, which resulted from major improvements in cloning, protein expression in heterologous systems, such as *Escherichia coli*, and protein purification by affinity chromatography, has significantly increased our ability to obtain the microgram to milligram quantities of protein needed for structure determination by X-­ray crystallography or NMR. Combined with genetic manipulation and engineering to facilitate heavy-atom integration into the target of interest, these developments greatly increased the range of proteins accessible to X-ray and NMR structure determination. Major techno­logical improvements in X-ray crystallography have significantly increased the resolution, speed and quality of data collection and structure determination. These developments included improved crystallization screening methods that enhanced our ability to obtain and optimize protein crystals, cryocooling techniques to obtain better quality data from single crystals and increased brightness, stability, availability and ‘user-friendliness’ of synchrotron and other X-ray sources. Together, these advances greatly enhanced the quality of data collection and extended it to smaller and smaller crystals. In parallel, advances in the ‘dry lab’, such as continued development and improvement of crystallographic and bioinformatics software packages, have increased the speed, reliability and quality of structure determination. Although it was clear that significant progress was being made in structural biology, it still paled in comparison to the escalating numbers of new protein sequences that were being generated by the worldwide DNA-sequencing efforts, such as the human genome project.

## 2. The Protein Structure Initiative

Exploration in to the concept of high-throughput (HT) protein-structure determination coincided with the dawn of the genomic era in biology. The enormous success of DNA-sequencing technology, followed by the development of other HT technologies, allowed biology to extend its molecular perspective to the level of entire organisms. These breakthroughs prompted global efforts into serious consideration of the feasibility of HT protein-structure determination, which included the establishment of pilot projects in the USA, Europe and Asia (Stevens *et al.*, 2001 ▶). In the USA, the NIGMS Protein Structure Initiative (PSI; http://www.nigms.nih.gov/Initiatives/PSI/) played a decisive role in embracing this new challenge in a timely way. The main scientific goal put forth by the PSI was to determine atomic level structures of most proteins readily obtainable from knowledge of their corresponding DNA sequences. Thus, structural biologists were enabled to broaden their focus from studying biological molecules one at a time and to a wider exploration of the rapidly expanding protein-sequence universe, thereby addressing questions on a genome scale. This new view of structural biology was, therefore, called structural genomics and added new perspectives and directions to the field (Stevens *et al.*, 2001 ▶).

**Figure 1:**
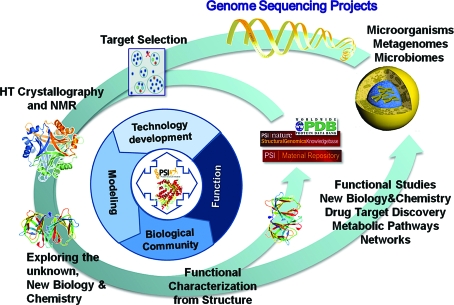
PSI Network strategy for structural genomics. In PSI-2, a network of research and resource centers were assembled in order to address its central mission of structural coverage of unexplored regions of protein-sequence space. Achieving a better understanding of the relationships between protein sequence and structure represents a critically important challenge to address the PSI’s principal goal of making structural information of most proteins readily available from knowledge of their corresponding gene sequences.

A technological revolution in structural biology then followed, which was in a large part propelled by the new worldwide structural genomics initiatives. Some might argue that this evolution would have happened in the absence of structural genomics, but there is no denying that these high-throughput initiatives had a significant impact on the field and greatly facilitated progress by creating large focused groups that could simultaneously optimize the myriad of complex steps in the structure-determination process. Assembling the initial gene-to-structure pipelines was rather challenging, but major advances and developments in technology, automation and method­ology were realised during the course of PSI-1 (2000–2005). The lessons gleaned from these efforts then enabled the assembly of high-throughput structural biology pipelines that developed into the Large-Scale Production (LSP) Centers in PSI-2 (2005–2010; Burley *et al.*, 2008 ▶) and provided the foundation for the PSI Network (http://www.sbkb.org/KB/psi_centers.html; http://www.nigms.nih.gov/Initiatives/PSI/Centers). The PSI-2 centers embraced the concept of broad structural coverage of protein-sequence space and vigorously pursued this goal by judicious and highly coordinated target selection that focused on selecting protein sequences that represented large families with little or no structural coverage and then leveraging the resulting experimental structures by homology modeling to cover other memebers of the protein family (Fig. 1 ▶).

During this period, the four LSP Centers expended the majority (70%) of their effort on these PSI Network targets. However, a significant effort (30%) was also committed to targets nominated by the broader research community and to specific biological/biomedical themes selected by each Center. As a result, the LSP Centers expanded and tailored their experimental and computational platforms to tackle a wide diversity of protein types and classes using a multitude of approaches, technologies and tools. They thereby identified a number of bottlenecks in the HT process for which solutions were achieved, thus opening new routes for solving the structures of many targets that had previously presented seemingly insurmountable obstacles. The six Specialized Centers in PSI-2 also continued the PSI tradition of developing innovative methods, approaches and technologies for protein production and structure determination of macromolecules and complexes that are considered to be highly challenging by the scientific community. The PSI Mater­ials Repository (PSI-MR) and the PSI/Nature Structural Genomics Knowledgebase (PSI-SGKB), now called PSI-SBKB in PSI:Biology were established in the latter half of PSI-2 in order to gather the valuable products of the PSI Centers and make them available to the general scientific community, and two Modeling Centers were formed to complete the PSI Network (http://www.nigms.nih.gov/Initiatives/PSI/Centers).

Collectively, the PSI Centers have accumulated a high level of expertise and knowhow in all steps from target selection to structure determination, developing pipelines that can be applied to many systems and to different classes of challenging proteins (Burley *et al.*, 2008 ▶). Many areas of science have already benefited significantly from the output of the LSP, as well from the methods and technologies developed by the Specialized Centers. Thus, the PSI has accelerated a wide range of basic research programs and provided new ideas and applications for the biomedical sciences. The critical mass of structural data generated by the PSI centers has also advanced our understanding of many biological processes. Going far beyond structure determination, the hundreds of thousands of reagents generated in the PSI are available through the PSI-MR for functional and mechanistic studies by the entire community.

The PSI is now entering its third phase as PSI:Biology, where investigators will apply this new paradigm of high-throughput structure determination to study a broad range of challenging biological and biomedical problems (http://www.nigms.nih.gov/Initiatives/PSI/psi_biology). The majority of the targets for structure determination will be defined through consortium partnership proposals that are vetted by peer review through the NIH grant system and *via* an open on-going community nomination process administered through the PSI-SBKB. Additional targets will be defined through the individual biological and biomedical theme projects of the LSP centers.

## 3. The JCSG: a scalable HT structural biology pipeline producing over 200 novel structures per year

### 3.1. Pilot phase

**Figure 2:**
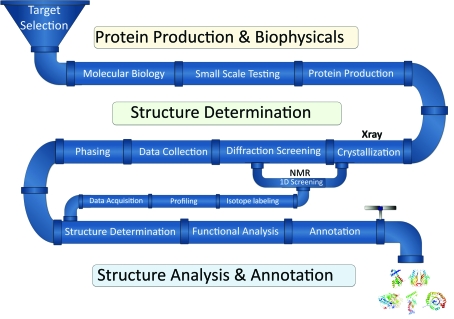
JCSG high-throughput structural biology pipeline. A linear representation is shown, highlighting the typical flow of targets through the multiple processing stages in the JCSG pipeline. The pipeline is subdivided into four main processing stages: (i) target selection, (ii) protein production and biophysical analysis, (iii) structure determination and (iv) structure analysis, annotation and distribution to public databases.

The Joint Center for Structural Genomics (JCSG; http://www.jcsg.org) was initiated in 1999 while the concepts of HT structural biology (HTSB) and structural genomics centers were still being formulated. At that time, most tasks associated with structure determination were highly labor-intensive, costly and could not be scaled to a genomic level without a substantial influx of resources and funding. The JCSG was established in 2000 as one of nine pilot centers under the auspices of the NIGMS PSI to evaluate the feasibility of assembling HT pipelines for protein-structure determination. The primary mission of the JCSG was to establish a robust and scalable HT structural biology pipeline and to assess its capability as a foundation for establishing production centers in PSI-2. The design, assembly and implementation of the JCSG pipeline was based on three scientific cores, each specializing in specific components of the gene-to-structure process: the BioInformatics Core (BIC; target selection and structure annotation), the Crystallomics Core (CC; cloning, protein production and crystallization) and the Structure Determination Core (SDC; crystal screening and structure determination). The assembly of these pipelines came from developing integrated and innovative technologies, methods and robotic platforms to circumvent bottlenecks in traditional structural biology. Our pipeline concept used a multi-tiered target-processing strategy that efficiently incorporated the multiple steps from target selection to structure deposition (Lesley *et al.*, 2002 ▶). The integration of these steps into a fully functional pipeline and its subsequent testing on the first screen of an entire genome (*Thermotoga maritima*; TM) stands as a unique accomplishment of the JCSG in PSI-1. The wide diversity of TM targets processed through the pipeline established success rates for individual steps in the process and enabled us to more reliably predict the outcome for each stage (Lesley *et al.*, 2002 ▶). The JCSG evolved to work effectively as a highly integrated team focused on the success of the overall pipeline and not just on its individual steps or components. Thus, over the course of PSI-1, the JCSG developed a fully integrated, scalable and high-output structural genomics pipeline which addressed both prokaryotic and eukaryotic targets (Fig. 2 ▶), including the incorporation of NMR as a pilot project to investigate the feasibility of equivalent high-throughput NMR approaches (Wüthrich, 2010 ▶). The pipeline currently utilizes X-ray structure determination for the majority of targets, whereas the NMR resources are reserved for targets that are not readily amenable to X-­ray structure determination, in addition to providing supplementary data such as screening protein stability and ligand binding.

### 3.2. Evolution of the HT pipeline

**Figure 3:**
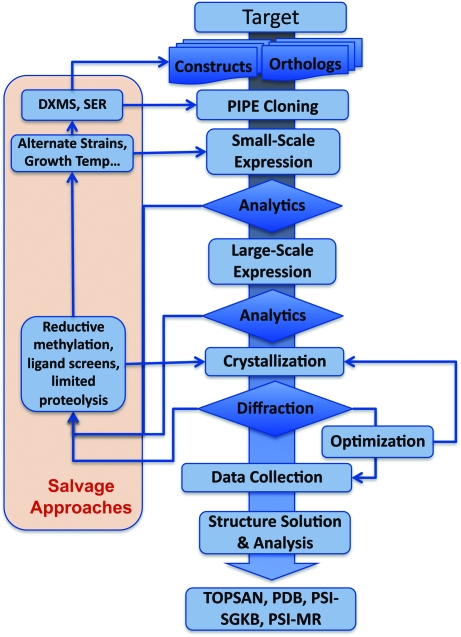
Evolution of main pipeline workflow. Flowchart of the current JCSG HTSB pipeline highlighting feedback loops and salvage pathways for recalcitrant targets.

The JCSG developed its HTSB pipeline in PSI-1 with an emphasis on scalability and adaptability that was driven by process data, basically demonstrating that executing an effective HTSB workflow is feasible. At the end of PSI-1, the JCSG was already producing 100 novel structures per year. In PSI-2, the LSP Centers were given a tripartite challenge to maximize output, minimize cost and greatly expand target diversity. The bar was also raised on the degree of difficulty of targets; proteins previously regarded as challenging in PSI-1 are now considered relatively routine. In PSI-2, the JCSG implemented HTSB on a production scale and further modified and extended it to reflect new production goals by anticipating challenges, by developing and adapting new technologies and by modifying workflow and resource allocations. The JCSG evolved to concomitantly increase pipeline efficiency, ramp up capacity and expand reach and, at the same time, significantly reduce operational costs compared to PSI-1. Midway through PSI-2, the JCSG had already significantly increased productivity and efficiency to achieve >200 structures per year while maintaining a high level of structural novelty. This success was mainly achieved from cumulative synergistically designed increases in efficiency at every stage, including more rational target selection that identified potentially problematic targets prior to the experimental stages. Small-scale bio-analytical screening of constructs, optimized structure solution, model building and validation, and the development of salvage pathways contributed to this increase in productivity and also increased the success with challenging targets (Fig. 3 ▶). Thus, the JCSG, along with the other LSP Centers, clearly demonstrated that parallel processing, miniaturization, statistical process analysis, standardization, efficient management structures, open data access and focused technology development can, indeed, be applied to front-line biological problems. Many of these principles and technologies have now been adopted worldwide, establishing their successful transfer to the community as a highly beneficial and influential outcome of the PSI.

While the PSI centers, including the JCSG, have not necessarily always been the initial inventors of some of the technologies or methodologies, they have demonstrated their general utility by further developing them and applying them to a very large number of targets so that meaningful statistics are now available on the success or failure of given methods, consequently offsetting the myriad of anecdotal stories that often have little predictive value. Thus, in developing our current pipeline, we tested experimental dogma and anecdotal truths, replacing them with well defined, reliable and well documented methods based on the analysis of thousands to tens of thousands of samples. These methods were then implemented as standardized HT approaches based on efficiency analyses. Our technology and innovation have been highly geared towards pipeline applicability and flexibility derived from cost/benefit analysis. For example, when it was determined that neither conventional restriction-enzyme cloning strategies nor newer ones, such as recombinatorial cloning, could meet our budget constraints and we needed to alter and insert DNA sequences with complete flexibility, the JCSG developed PIPE cloning (Klock *et al.*, 2008 ▶). This advance permitted the insertion or modification of genes in virtually any vector system with complete flexibility. Table 1 ▶ lists some other important parameters for the successful development of the JCSG Crystallomics pipeline during PSI-1 and PSI-2. The JCSG has recently embarked on the next phase, PSI:Biology, in which the technologies and methods developed and refined during the previous phase will be applied to challenging biological systems in association with our PSI:Biology partner centers.

### 3.3. HT pipeline

**Figure 4:**
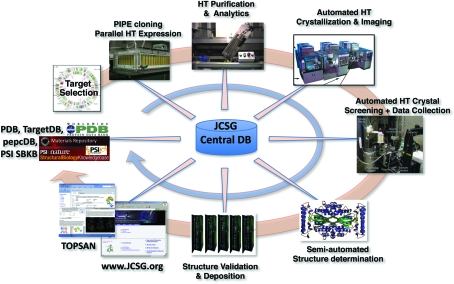
JCSG PSI-2 production pipeline and underlying data-handling architecture. The JCSG large-scale production center integrates custom and commercial instrumentation into the highly parallelized HTSB pipeline. Data capture from >500 parameters encompassing over 30 stages are captured to a centralized database *via* the JCSG tracking database that parallels the experimental pipeline. Data flow back to the experimental pipeline provides feedback for target and pipeline management and smart target selection.

Parallel processing and data capture is a primary driver of the JCSG pipeline and is incorporated into all stages of the process (Fig. 4 ▶). Targets enter the pipeline either from the PSI Network, our own biomedical/biological theme projects or from the community and are subjected to computational filters that estimate the probability of success of a given target. Each target then proceeds to the cloning stage, accompanied by a set of complementary homologs and/or constructs, resulting in multiple, full-length clones or truncated constructs, with the goal of increasing our chance of success in later stages. Each of these constructs is then evaluated by microexpression, which produces sufficient protein for extensive biophysical characterization, yielding an experimental estimate of the likelihood of crystallization success. Thus, the pipeline efficiently produces vast collections and varieties of proteins for crystallization trials that result in an equally impressive number of crystals to screen for the best diffracting samples. High-quality diffraction data enable structures to be refined and validated to exacting specifications before deposition in the PDB, with independent, internal, quality-control procedures designed to maintain a uniformly high quality of the deposited structures. Vectors and clones are deposited with the PSI-MR and resources, methods, analyses *etc*. are entered into the PSI-SBKB. Targets are tracked from start to finish in the internal tracking database available through the JCSG website (http://www.jcsg.org), with the most pertinent information exported to TargetDB (http://targetdb.pdb.org). Finally, information on all experimental protocols is deposited in PepcDB (http://pepcdb.pdb.org), and TOPSAN (Krishna *et al.*, 2010 ▶; Weekes *et al.*, 2010 ▶) pages provide public access and outreach to the multiple diverse analyses on this enormous resource of protein structures.

## 4. Concluding remarks

Structural genomics is now established as a field that is making major contributions to our knowledge of the protein-sequence universe (Nair *et al.*, 2009 ▶). The genome-sequencing efforts have provided structural genomics with unheralded opportunities to explore the richness and diversity of life forms and the fundamental processes that allow organisms to evolve and function in their own niches and environments. The development of HT platforms from target selection to structure determination has enabled structural genomics to embrace these new exciting challenges and opportunities. As we move forward into PSI:Biology, we will explore how these efficient and highly productive HT pipelines can be harnessed not only to tackle some of the most prescient and topical problems in the biological and biomedical sciences, but also to create exciting new research areas for future generations of scientists. Many possible strategies employing the HTSB technologies and platforms can be envisioned, ranging from general exploration of the expanding protein universe to more focused forays into individual organisms, protein networks, pathways or specific families of proteins.

This special JCSG issue of *Acta Crystallographica Section F* aims to give a flavor of the type and range of projects that we have been working on over the last few years and illustrates some of the insights that can be gained from structural genomics. The JCSG has recently passed the 1000-structure milestone, where the majority of the structures are novel (>80%) as defined by a less than 30% sequence identity of the JCSG structure to any other structure in the PDB at the time of deposition. The structures deposited range from bacterial to human proteins, although the vast majority are of bacterial origin, since these were the ones on which the pipeline was assembled and tested. The large numbers of targets and the enormous amounts of associated data collected and processed through the multiple stages of our experimental pipeline have resulted in the development of innovative methods and tools at critical stages in our gene-to-structure pipeline. These resources, where feasible, have been converted to free-access web-based tools and applications that include *XtalPred* (http://ffas.burnham.org/XtalPred; Slabinski, Jaroszewski, Rychlewski *et al.*, 2007 ▶), Quality Control (http://smb.slac.stanford.edu/jcsg/QC) and Ligand Search (http://smb.slac.stanford.edu/jcsg/Ligand_Search; Kumar *et al.*, 2010 ▶) servers and TOPSAN (http://www.topsan.org; Krishna *et al.*, 2010 ▶; Weekes *et al.*, 2010 ▶), as well as other tools and resources available on our website (http://www.jcsg.org). In particular, the JCSG maintains a crystallographic data-set repository that contains all files from all stages of the structure-solution process, including a full set of diffraction images for each of our deposited structures, enabling complete reconstruction of the data processing (http://www.jcsg.org/datasets-info.shtml). The repository has been used by numerous prominent crystallographic software developers to assist in testing new algorithms (Yao *et al.*, 2006 ▶; Cowtan, 2008 ▶; Panjikar *et al.*, 2009 ▶; Sauter & Zwart, 2009 ▶; Skubák *et al.*, 2009 ▶; Terwilliger *et al.*, 2009 ▶; Winter, 2005 ▶) and has also been used for benchmarking (Joosten *et al.*, 2009 ▶) and teaching (Faust *et al.*, 2008 ▶). Individual data sets can also be downloaded directly from the JCSG structure gallery (http://www.jcsg.org/prod/newscripts/structure_gallery/gallery.cgi). The repository currently has 72 registered users, many of whom are students or postdocs who are keen to have access to data sets to advance their knowledge of X-ray crystallography. The repository is freely available to the scientific community and will be maintained and extended in PSI:Biology. We understand that such resources are of high value to the general scientific community and we welcome any feedback and comments on how to improve their utility.

As we move forward, the goal is to continue to push the limits of what can be accomplished in structural biology and to ensure that the methods, technologies and automation that have been and are being developed are transferable to individual laboratories (Gräslund *et al.*, 2008 ▶). Many of the advances at synchrotron beamlines have occurred in partnership with Structural Genomic centers and likewise many of the available crystallization robots and protein expression and purification devices and platforms, as well as software packages and tools, have their origin in the PSI and other SG centers. The following articles give a tantalizing glimpse of the exciting possibilities for the future, but also highlight the need to ensure that concomitant advances in other basic tools are also realised in order to enable exploration of the functional ramifications of the impressive crop of new structures. A vast number of the genes still have no assigned function and it is hoped and encouraged that major efforts to accelerate discovery of function will now follow as a result of the resounding success in HT gene sequencing and HT structure determination. The new glimpses into protein-structure evolution and the insights into structural and functional diversification of proteins that we have gained through the large-scale protein structure determination gives us a preview of what further new discoveries could be unearthed by novel applications of HT structure determination.

## Figures and Tables

**Table 1:** JCSG pipeline economic drivers

Parameter	Impact (related publication)
Nanovolume crystallization	Significant reduction in the quantity of protein and reagents per screen; faster results (Weselak *et al.*, 2003 ▶; Santarsiero *et al.*, 2002 ▶)
PIPE cloning	(Klock *et al.*, 2008 ▶; Spraggon *et al.*, 2004*a* ▶)
Orthologs, mutations, truncations	(Klock *et al.*, 2008 ▶; Spraggon *et al.*, 2004*b* ▶, 2005 ▶)
Optimized crystal screens	Minimal conditions to screen (Page *et al.*, 2003 ▶)
Parallel processing	Unit cost reduction, efficient workflow (DiDonato *et al.*, 2004 ▶; Lesley *et al.*, 2002 ▶)
Optimized salvage	Increased output at minimal cost
Automation	Speed, consistency, decreased FTE costs (Lesley *et al.*, 2002 ▶; McPhillips *et al.*, 2002 ▶; Wolf *et al.*, 2005 ▶; Weselak *et al.*, 2003 ▶; Soltis *et al.*, 2008 ▶)
Smart target selection	Increased per target success rate (Slabinski, Jaroszewski, Rodrigues *et al.*, 2007 ▶), genome pool strategy (Jaroszewski *et al.*, 2008 ▶)
